# Mental health and sleep quality issues in adolescents with chronic conditions during and after COVID-19 quarantine

**DOI:** 10.1016/j.clinsp.2024.100364

**Published:** 2024-10-15

**Authors:** Renan Andrews de Sousa, Levi Medeiros Vieira Paradelas, Livia Lindoso, Reinan Tavares Campos, Rafaela Mendes Battiferro, Beatriz Oliveira Leão Carneiro, Jean Paulo Veronesse de Souza, Marianna Ribeiro de Menezes Freire, Maria Paula Ribeiro Cardoso, Claudia Alejandra Ayala Strabelli, Clovis Artur Silva

**Affiliations:** Instituto da Criança e Adolescente, Hospital das Clínicas da Faculdade de Medicina da Universidade de São Paulo (HCFMUSP), São Paulo, SP, Brazil

The COVID-19 pandemic was considered a catastrophic event for physical and mental health in adolescents.^1^^,^^2^ A systematic review showed an increased incidence of psychiatric issues for this population during this period,^3^ and the World Health Organization (WHO) also categorized adolescents with chronic conditions as a high-risk group during the pandemic.^4^

The authors demonstrated in a cross-sectional study that one-third of both chronic immunocompromised conditions and healthy teens had severe psychosocial dysfunction during COVID-19 quarantine. Female sex, fear of underlying disease activity or complication, and household members working outside of the home were relevant factors associated with adolescents with chronic immunocompromised conditions.^5^ The authors also showed poor sleep quality frequencies in both groups (38 % and 48 %) in another report.^6^ Another cross-sectional study of our group identified emotional, hyperactivity and inattention problems in up to one-third of immunocompromising chronic disease teens during this emergent period.^7^ All these researches were performed before the COVID-19 vaccination period for adolescents.^5^, ^6^, ^7^

A prospective study evaluated emotional issues during the COVID‐19 pandemic in Brazil, and showed fluctuations in mental symptoms of non-vaccinated children and adolescents. Lowest socio-economic status, chronic condition demanding treatment and family members with COVID-19 have increased these mental issues over time.^8^

A recent meta-analysis study showed that the prevalence of mental health issues increased during the COVID-19 pandemic progression, even though recovery and stabilization of these were also evidenced. However, this report did not include subgroup analysis of immunocompromising chronic conditions, precluding a definitive conclusion of longitudinal mental health impact for this population.^9^ Therefore, the objective of this prospective study was to evaluate mental health and sleep quality issues in the teen population in two distinct moments: during the COVID-19 pandemic (non-vaccinated adolescents) and after the COVID-19 pandemic (vaccinated adolescents).

Between June and December 2022, a follow-up online survey was sent for the same subject's population (*n* = 504) of previous studies,^5^, ^6^, ^7^ using the validated instruments to assess prospectively: mental health (Strengths and Difficulties Questionnaire [SDQ]), Health-related quality of life (HRQL) (Pediatric Quality of Live Inventory 4.0 [PedsQL4.0]) and sleep quality [Pittsburgh Sleep Quality Index (PSQI)]. The survey was sent using the REDCAP© platform at least six times to each subject, and contained a consent form signed by the guardians, an assent form signed by the adolescents, a semi-structured socioeconomic questionnaire, and the validated instruments.

During recruitment, *n* = 38 (35.2 %) of healthy adolescents and *n* = 86 (25.1 %) patients signed assent form. However, only *n* = 27 participants (6.0 %) (*n* = 18 patients and *n* = 9 healthy controls) finished the follow-up survey and had previously answered the questionnaires during the COVID-19 pandemic. According to diagnosis, rheumatologic conditions occurred in *n* = 4 (22.2 %), gastrointestinal conditions in *n* = 8 (44.4 %), and renal conditions in *n* = 6 (33.3 %).

Further comparison between adolescents during versus after COVID-19 quarantine showed that vaccinated against COVID-19 (≥ 2 shots; 0 vs. 96 %, *p* = 0.0001), vaccinated against COVID-19 (3 shots; 0 vs. 55 %, *p* = 0.001) and COVID-19 diagnosis confirmed by test (0 vs. 41 %, *p* = 0.0003) were significantly higher in the former group. Online school activities were significantly reduced after COVID-19 quarantine (*p* < 0.0001) (Table 1).

**Table 1:** Demographic data, Strengths and Difficulties Questionnaire (SDQ), Pittsburgh Sleep Quality Index (PSQI) and Pediatric Quality of Live Inventory 4.0 (PedsQL4.0) scores reported by adolescents during COVID-19 quarantine and after COVID-19 quarantine.

**Domains**	**Adolescents during COVID-19 quarantine** **(*n* = 27)**	**Adolescents after COVID-19 quarantine** **(*n* = 27)**	**p**
**Semiestructured socioeconomical questionaire**			
**Demographic data**			
Current age	14 (10‒17)	16 (12‒19)	**0.001**
Female sex	13 (48)	13 (48)	1.000
**Use of daily medication**	17 (63)	13 (50)	0.500
**Psychological or psychiatric care**	2 (7)	7 (26)	0.142
**COVID-19 diagnosis and vaccination**			
Vaccinated against influenza	16 (59)	19 (70)	0.393
Vaccinated against COVID-19 (≥ 2 shots)	0 (0)	26 (96)	**0.0001**
Vaccinated against COVID-19 (3 shots)	0 (0)	15 (55)	**0.0001**
COVID-19 diagnosis confirmed by test	0 (0)	11 (41)	**0.0003**
Hospitalization due to COVID-19	0 (0)	3 (11)	0.236
**Sleep time**			
Sleep after midnight	19 (70)	14 (52)	0.163
Sleep before midnight	8 (30)	13 (48)	
**Sleeping disorder**	9 (33)	7 (26)	0.551
**Online school activities**			
Without	0 (0)	17 (63)	**<0.0001**
≥ 3 h/day	14 (52)	5 (19)	
< 3 h/day	13 (48)	5 (18)	
**Housework**			
None	7 (26)	7 (26)	0.788
≤1 hour/day	5 (18)	7 (26)	
>1 hour/day	15 (56)	13 (48)	
**Screen time**			
≤ 2 h/day	0 (0)	3 (11)	0.134
3‒6 h/day	16 (59)	11 (41)	
> 6 h/day	11 (41)	13 (48)	
**Family financial status**			
Did not change	19 (70)	17 (63)	0.820
Worsen	7 (26)	8 (30)	
Improved	1 (4)	2 (7)	
**SDQ**			
**SDQ score**			
SDQ total score (0‒40)	12.7 ± 6.3	11.6 ± 5.8	0.350
Abnormal SDQ, (total score 20‒40)	5 (18.5)	4 (14.8)	0.715
**SDQ domains scores**			
Prosocial (0‒10)	7.04 ± 2.3	7.37 ± 2.0	0.431
Hyperactivity (0‒10)	4.41 ± 3.0	4.04 ± 2.7	0.464
Emotional (0‒10)	3.48 ± 2.5	3.48 ± 2.5	1.000
Conduct (0‒10)	2.52 ± 2.0	1.85 ± 2.0	0.077
Peers (0‒10)	2.30 ± 1.8	1.85 ± 2.0	0.389
Internalizing (0‒20)	5.78 ± 3.6	5.97 ± 3.6	0.877
Externalizing (0‒20)	6.93 ± 4.4	5.89 ± 3.9	0.148
**SDQ domains abnormal scores**			
Prosocial disorders, (score 0‒4)	11 (40.7)	8 (29.6)	0.393
Hyperactivity disorder, (score 7‒10)	8 (29.6)	5 (18.5)	0.340
Emotional disorder, (score 7‒10)	5 (18.5)	5 (18.5)	1.000
Conduct disorder, (score 5‒10)	5 (18.5)	4 (14.8)	1.000
Peers problems, (score 6‒10)	5 (18.5)	4 (14.8)	1.000
Internalizing disorder, (score 11‒20)	5 (18.5)	5 (18.5)	1.000
Externalizing disorder, (score 13‒20)	6 (22.2)	4 (14.8)	0.484
**PSQI score**			
**PSQI score**			
PSQI total score (0‒21)	5.65 ± 2.6	3.48 ± 3.0	**0.038**
Good sleep quality, total score ≤5	9 (39.1)	20 (70.4)	**0.013**
**PSQI domains scores**			
Overall sleep quality (0‒3)	0.69 ± 0.88	0.74 ± 0.62	0.285
Sleep latency (0‒3)	1.13 ± 0.97	0,91 ± 0.90	0.347
Sleep duration (0‒3)	0.13 ± 0.63	0.26 ± 0.54	0.479
Sleep efficiency (0‒3)	0.50 ± 0.89	0.88 ± 1.26	0.304
Sleep disturbances (0‒3)	1.26 ± 0.45	1.17 ± 0.39	0.328
Sleep medication use (0‒3)	0.78 ± 1.24	0.61 ± 1.20	0.590
Day dysfunction due to sleepiness (0‒3)	0.96 ± 0.93	0.83 ± 1.15	0.613
**PedsQL4.0 score**			
**Total score (0‒100)**	77 (35‒95)	77 (44.5‒100)	0.440
**Physical health summary score (0‒100)**	84 (31‒100)	81 (38‒100)	0.792
**Psychosocial health summary score (0‒100)**	73 (20‒92)	78 (45‒100)	0.203
Emotional functioning (0‒100)	65 (30‒95)	75 (25‒100)	0.114
Social functioning (0‒100)	93 (20‒100)	90 (65‒100)	0.715
School functioning (0‒100)	65 (0‒100)	75 (15‒100)	0.189

Results are presented in median (minimum‒maximum values), mean ± standard deviation and n (%).

Importantly sleep quality of adolescents improved after the COVID-19 quarantine. The mean PSQI total score was significantly higher in adolescents during versus after COVID-19 quarantine (5.65 ± 2.6 vs. 3.48 ± 3.0, *p* = 0.038), whereas the frequency of good sleep quality was significantly lower before COVID-19 quarantine (39.1 vs. 70.4, *p* = 0.013).

Abnormal SDQ score was high and similar in both groups (18.5% vs. 14.8 %, *p* = 0.715). No differences were observed before and after COVID-19 quarantine in all parameters of mental health score (SDQ) and HRQL (PedsQL) (*p* > 0.05).

The main limitation of the present study was low adherence to questionnaire responses. This finding may be related to tiredness, possibly due to fatigue by the information overload of the COVID-19 quarantine. Low response rates in research also have been described during SARS-CoV-2^10^ and may be related to subjects feeling undervalued^,^ and media overexposed of adolescents during and after this emergency crisis.

In conclusion, the present study showed psychosocial dysfunction in approximately 15 % of adolescents with chronic immunocompromised conditions and healthy teens after the COVID-19 pandemic, with longitudinal improvement in sleep quality. This report highlights the necessary implementation of future programs to protect the mental health of teens from other public health disasters or emergencies.

## Declaration of competing interest

The authors declare no conflicts of interest.
